# Metabolomic analyses provide insights into the preharvest rind disorder in Satsuma Owari Mandarin

**DOI:** 10.3389/fpls.2023.1263354

**Published:** 2023-09-26

**Authors:** Tariq Pervaiz, Suejin Park, Alaaeldin Rezk, Manhoi Hur, David Obenland, Mary Lu Arpaia, Ashraf El-kereamy

**Affiliations:** ^1^Department of Botany and Plant Sciences, University of California, Riverside, Riverside, CA, United States; ^2^Department of Horticulture, Jeonbuk National University, Jeonju, Republic of Korea; ^3^Metabolomics Core Facility, Institute for Integrative Genome Biology, University of California, Riverside, Riverside, CA, United States; ^4^United States Department of Agriculture (USDA), Agricultural Research Service, San Joaquin Valley Agricultural Sciences Center, Parlier, CA, United States

**Keywords:** mandarin, rind physiological disorder, non-chilling rind disorder, metabolites, antioxidants, hormones

## Abstract

Citrus fruit’s appearance is the primary criterion used to assess its quality for the fresh market, hence the rind’s condition is a crucial quality trait. Pre-harvest rind disorder is one of the major physiological problems in mandarins. The disorder occurs right before harvest following rain events in some Mandarin varieties. Despite the economic damage caused by this kind of disorder, very limited information is available about the molecular mechanisms underlying the occurrence of this disorder. In the present study, we evaluated the primary metabolites, antioxidants, and hormones associated with the pre-harvest rind disorder in Mandarins. The study was carried out using ten-year-old ‘Owari’ Satsuma mandarin trees grafted on ‘Carrizo’ rootstock and grown in a commercial orchard in San Joaquin Valley, California, USA. Samples were collected from healthy tissue of healthy fruit (HF_HT), healthy tissue of damaged fruit (DF_HT), and damaged tissue of damaged fruit (DF_DT). Damaged fruit (DF_HT and DF_DT) showed lower cellulose concentrations than healthy fruit tissues (HF_HT), however, had similar contents of pectin and hemicellulose. The antioxidant activities showed no significant difference in all paired comparisons between samples as expressed in the malondialdehyde (MDA) content. However, DF_DT had a higher H_2_O_2_ content compared to HF_HT, but DF_HT had a similar content to that of HF_HT. Furthermore, peroxidase (POD) and polyphenol oxidase (PPO) activities were increased in DF_DT compared to HF_HT (*P* = 0.0294) and DF_HT (*P* = 0.0044), respectively. Targeted metabolomics analysis revealed that a total of 76 metabolites were identified in Satsuma rind tissues, and the relative concentrations of 43 metabolites were significantly different across studied samples. The hormonal analysis showed the involvement of jasmonate O-methyltransferase, jasmonic acid-amido synthetase JAR1-like, and JA-isoleucine may key role in causing the rind disorder in mandarins. In addition, the damaged fruit tissues have a higher level of jasmonic acid (JA), 12-oxo-phytodienoic acid, and JA-isoleucine than undamaged tissue.

## 1 Introduction

Appearance is the primary parameter used to assess fruit quality for the fresh market, hence the rind condition is a crucial quality trait. Rind disorders result from various issues including insects, pathogens, chilling, and non-chilling rind damage. The limited understanding of the physiological mechanisms underlying these disorders has an impact on the commercial value of citrus. The varied preharvest, harvest, and postharvest environments to which citrus fruit is subjected are thought to have an impact on physiological rind problems. The concept behind most fruit physiological problems links preharvest, eco-physiological, and postharvest factors (Cronjé, 2007; Assimakopoulou et al., 2009; Magwaza et al., 2014). One of the main difficulties in post-harvest management is preventing the development of physiological rind disorders that don’t require freezing, such as rind disintegration, preharvest chilling disorder, rind staining, rind puffiness, and peteca spots. Intensive research has been conducted toward a better understanding of the factors contributing to the incidence of these disorders (Magwaza et al., 2014). Despite the fact that these physiological abnormalities only affect the fruit’s rind and do not damage its edible content, they do reduce post-harvest fruit market value because of poor physical appearance and the resulting consumer complaints (Alquezar et al., 2010; Magwaza et al., 2014).

The epidermis of plants and fruits is frequently exposed to pathogenic and non-pathogenic microorganisms in the environment. Consequently, the cuticle is essential in the interaction with these microorganisms in addition to serving as a protective barrier (Lafuente et al., 2021). Most physiological disorders are theoretically associated with preharvest, eco-physiological, and postharvest factors (Witney et al., 1990; Magwaza et al., 2014). The major citrus growing areas in California often have preharvest non-chilling rind disorder in mandarins that appears following rainwater sticking to the fruit surface. Adaskaveg et al. (2010) reported the same problem in the various citrus-growing counties in California, from the southern to northern Central Valley (e.g., Butte, Fresno, Kern, and Tulare Counties), outbreaks of a mandarin rind condition that led to the significant loss of citrus crops. Furthermore, previously the same problem was reported in various citrus fruits including Valencia and navel oranges (Chapman, 1958; Adaskaveg et al., 2010). Until now valuable evidence and reliable data are not available and reports are not conclusive. As a result, the disorder has not yet been documented with a specific name and etiology; herein, we referred to it as a preharvest non-chilling rind disorder.

Preharvest factors that affect rind condition have recently gained interest since many fruit disorders are intimately connected to later stages of fruit ripening and do not necessarily require certain conditions to develop. Different varieties of fruit have varying susceptibilities to a physiological rind disorder. The concentration of several phytohormones in the ‘Nules Clementine’ mandarin fruit rind during postharvest non-chilling cold storage demonstrates that the fruit contains these hormones (Olarewaju et al., 2022). Additionally, citrus fruits that are kept in the cold during storage respond to heat significantly depending on the pre-harvest environment. This triggers the phenylalanine ammonia-lyase production, which is the first step in phenylpropanoid metabolism, increase in most fruits for a given chilling injury index (Sala and Lafuente, 2004; Lafuente et al., 2017; Medda et al., 2020). Physiological studies have demonstrated the importance of hormones, oxidative stress, lipids, carbohydrates, and phenolic metabolism in the susceptibility of citrus fruits to chilling as well as in the development of heat-induced chilling tolerance (Lafuente et al., 2005). Reactive oxygen species (ROS) produced under stressful conditions, such as exposure to ozone, cold, UV radiation, and water stress, have been linked to the degradation of plant tissues (Sala and Lafuente, 2004; Medda et al., 2020). Acyl lipid peroxidation, which is mediated by hydroxyl radical generation, is strongly related to the considerable alteration of the membrane composition in pitted tissues. It was determined that the fatty acids in pitted tissues had not fully degraded. Through the release of oxidative products, tonoplast disruption in damaged cells hastened the spread of disorder to neighboring cells, preventing organelles and membranes from functioning normally (Filipe Vitor et al., 2001; Magwaza et al., 2014). An alternative significant contributing factor to rind disorder in Satsuma mandarin is thought to be phytohormones. Exogenous GA_3_ application is believed to cause rind disorder at an early stage of fruit development (Lu et al., 2017). Regardless of the water potential in the epidermis of fruits, the water on the surface, as mentioned above with citrus rind disorder, may cause oxidative damage to tissue and block the gas exchange from the fruit epidermis and reduce metabolic activities.

In the current study, we collected Satsuma mandarin fruits with and without preharvest rind disorder in several orchards in central California. In mandarin fruits, the genes associated with the rind disorder and their molecular mechanism are not clear. To bridge this knowledge gap, the metabolomic mechanisms involved in rind disorder, we conducted a comprehensive metabolomic and hormonal analysis in healthy and damaged fruits in order to better understand the cause and control of rind disorder.

## 2 Materials and methods

### 2.1 Plant materials and sampling

**Figure 1:**
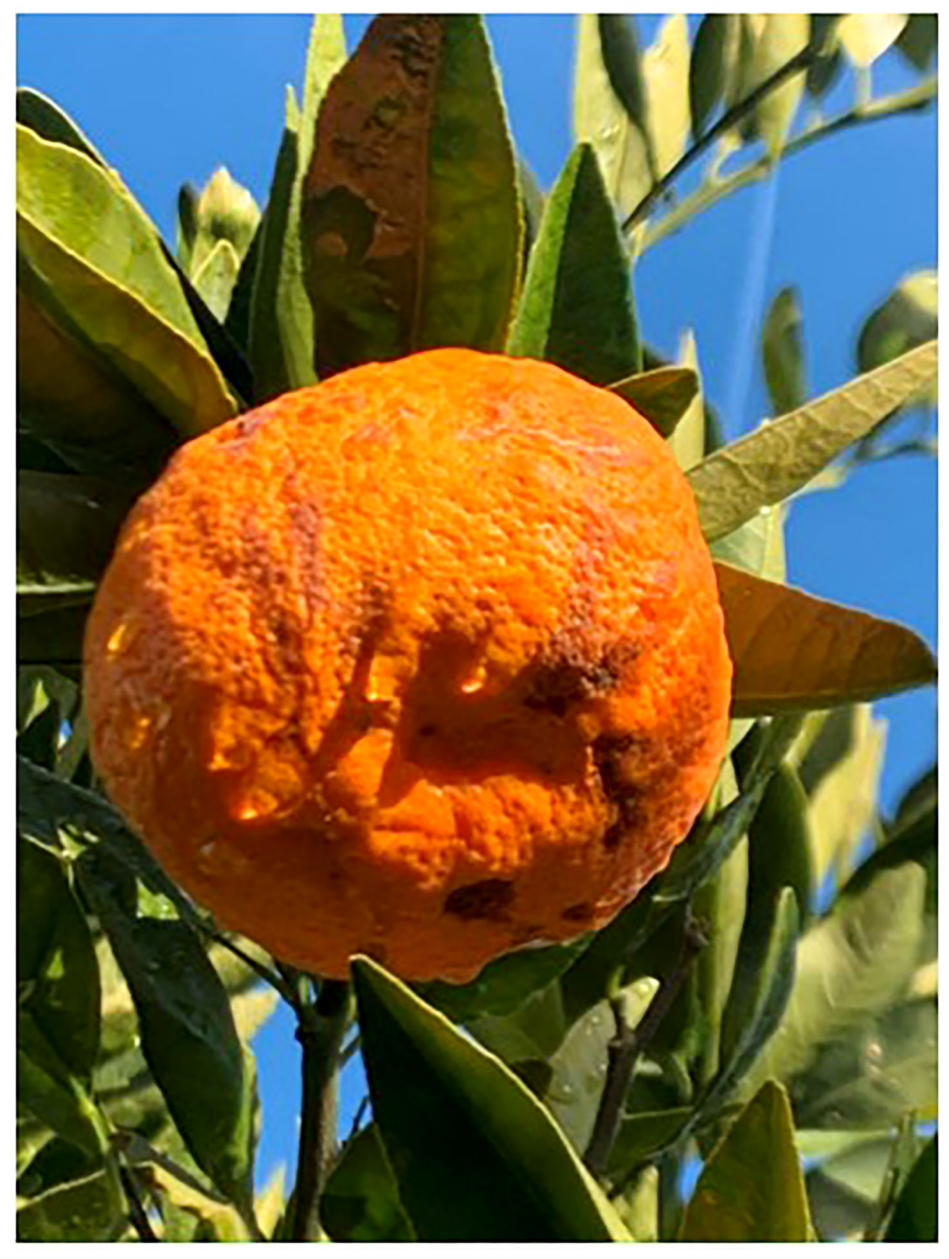
Pre-harvest rind disorder in Satsuma mandarin (C. unshiu Marc. ‘Owari’).

The fruit samples were randomly collected from the outer part of the tree canopy from ten-year-old Satsuma ‘Owari’ mandarin trees during the 2019 season (**Figure 1**). The analysis was conducted with four replicates, where the tissue of each replicate was collected from four fruits harvested from the outer canopy of distinct trees. Tissues were separated as follows: healthy tissue of healthy fruit (HF_HT), healthy tissue of damaged fruit (DF_HT), and damaged tissue of damaged fruit (DF_DT) (**Supplementary Figure 1**). The tissues were collected from the harvested fruits and were immediately frozen in liquid nitrogen and stored at -80°C until used for further analyses. Tissues were ground in a Qiagen TissueLyser (Qiagen Group USA) with liquid nitrogen and stored at -80°C.

### 2.2 Cell wall composition analysis

Three grams of ground rind tissues were homogenized with 20 mL of 95% ethanol and boiled for 30 min to inactivate cell wall modifying enzymes. The homogenate was centrifuged at 15,000 × g for 5 min at room temperature (RT), then the supernatant was discarded. The residue was added to 20 mL of 95% ethanol and centrifuged at the same conditions. The residue was subsequently washed with 20 mL of chloroform: methanol (1:1) mixture and air-dried overnight, yielding the alcohol-insoluble residue (AIR).

Fractions of different cell wall components were analyzed by sequential extractions of the AIR as previously described with minor modification (Fangel et al., 2021). Forty milligrams of AIR were suspended in 10 mL of water and incubated at 95°C for 10 min, centrifuged at 6,000 × g for 5 min at 4°C, and filtered on Whatman™ 54 filter paper (Cytiva, Marlborough, MA, USA). The filtrate was taken to 11 ml with distilled water and designated as a water-soluble fraction (WSF). The residue was then placed in 10 mL of 50 mM trans-1,2-diaminocyclohexane-tetraacetic acid (CDTA) for 3 hr. at RT. The slurry was centrifuged, filtered, and the filtrate was collected as described above. The extract was designated as a CDTA-soluble fraction (CSF). The CDTA-insoluble pellet was then extracted with 10 mL of 50 mM Na_2_CO_3_ at 4°C for 1 hr. After centrifugation and filtration, the extracted solution was designated as Na_2_CO_3_-soluble fraction (NSF). Subsequently, the pellet was extracted with 10 mL of 1 M KOH at 4°C for 1 hr. The extract was designated as 1 M KOH-soluble fraction (1KSF). Finally, the pellet was re-extracted with 4 M KOH for a 4 M KOH-soluble fraction (4KSF). The residue was washed three times with ethanol 50% (v/v), dried at 65°C, and designated as α-cellulose.

Uronic acid contents from WSF, CSF, and NSF were measured as pectin contents according to Blumenkrantz and Asboe-Hansen (1973). Aliquots of each cell wall fraction were taken to 20 µL with distilled water. Subsequently, 200 µL of 98% (w/w) H_2_SO_4_ containing 75 mM sodium borate was added and the mixture was placed into a water-ice bath. The mixture was shaken and incubated at 95°C for 10 min. After cooling down in a water-ice bath, 5 µL of 0.15% (w/v) m-phenyl phenol in 0.5% (w/v) NaOH was added. The absorbances at 520 nm were measured in a UV/Vis spectrophotometer (accuSkan™ GO UV/Vis Microplate Spectrophotometer; Thermo Fisher Scientific, Inc., Waltham, MA, USA). The standard curve was generated using galacturonic acid in the range of 0-50 µg·mL^-1^.

The Anthrone method was used to measure neutral sugars for hemicellulose from 1KSF and 4KSF (Yemm and Willis, 1954). Aliquots from each cell wall fraction were diluted to 100 µL with distilled water. Subsequently, 200 µL of 2 g·L^-1^ anthrone (in 98%, w/w H_2_SO_4_) was added to a water-ice bath. The samples were then incubated for 10 min at 95°C. The reaction mixtures were cooled in a water-ice bath and the absorbances at 620 nm were measured using a UV/Vis spectrophotometer. The standard curve was established using glucose in the range of 0 – 300 µg·mL^-1^.

### 2.3 Determination of lipid peroxidation

Lipid peroxidation in rind tissue was estimated as contents of total 2-thiobarbituric acid (TBA) reactive substances and expressed as equivalents of malondialdehyde (MDA) according to Aguilar Diaz De Leon & Borges (2020). One hundred milligrams of ground tissues were homogenized with 1.6 ml of 0.1% trichloroacetic acid (TCA) solution and centrifuged at 12,000 × g for 15 min at 4°C. The 0.5 ml of the supernatant was reacted with 1.5 ml of 0.5% TBA in 20% TCA for 30 min at 95°C. The reaction was then cooled down in a water-ice bath. The samples were centrifuged at 9,000 × g for 10 min at 4°C. The specific and non-specific absorbances were measured at 532 and 600 nm, respectively. The non-specific absorbance was subtracted from the specific absorbance, and an extinction coefficient of 155 mM^-1^·cm^-1^ was used to calculate the MDA concentration.

### 2.4 Determination of hydrogen peroxide contents

One hundred milligrams of ground tissue were added to a reaction mixture with 0.1% TCA and 1M potassium iodide in 10 mM potassium phosphate buffer (pH 7.0). After 10 min incubation at 4°C, the homogenate was centrifuged at 12,000 × g for 15 min at 4°C. The absorbance of the supernatant was measured at 350 nm using a UV/Vis spectrophotometer. The standard curve was generated with H_2_O_2_ standard solutions.

### 2.5 Determination of antioxidant activity

Ground tissue (100 mg) was homogenized in 1 ml of 70% methanol by stirring for an hour at RT in the dark. The suspension was centrifuged at 3,000 × g for 10 min at 4°C. The supernatant was transferred to a new tube and stored at -20°C. One milliliter of 70% methanol was added into the original tube and the extraction was repeated as described above. The second supernatant was combined with the first supernatant. The extract (10 µl) was added with 190 µl of 200 µM 2,2-diphenyl-1-picrylhydrazyl (DPPH) solution, and then incubated at RT for 30 min. The absorbance of DPPH free radical was measured at 515 nm with a UV/Vis spectrophotometer.

### 2.6 Antioxidant enzyme assay

One hundred milligrams of ground tissue were homogenized in 1.7 ml potassium phosphate buffer (50 mM, pH 7.0) containing 1 mM EDTA for 1 min. The suspension was centrifuged at 15,000 × g for 20 min at 4°C. The supernatant was collected for subsequent measurements of superoxide dismutase (SOD), peroxidase (POD), and polyphenol oxidase (PPO).

Superoxide activity was assayed by monitoring the inhibition of the photochemical reduction of nitroblue tetrazolium (NBT). In brief, 1.5 ml reaction mixtures comprising 50 mM potassium phosphate buffer (pH 7.0), 13 mM methionine, 75 µM NBT, 2 µM riboflavin, 1 mM EDTA, and 50 µl enzyme extract were illuminated for 20 min under a fluorescent light, and then the absorbance was measured at 560 nm using a UV/Vis spectrophotometer.

The activity of POD was analyzed by adding 20 µl aliquots of tissue extract to a 100 µl reaction mixture containing 50 mM potassium phosphate buffer (pH 7.0), 3 mM guaiacol, and 5 mM. Increases in absorbance were measured at 470 nm for 3 min at 25°C using a UV/Vis spectrophotometer. An extinction coefficient of 6.39 mM^-1^·cm^-1^ was used to calculate the H_2_O_2_ concentration. One unit of POD activity was equivalent to 1 mM of H_2_O_2_.

Polyphenol oxidase activity was measured by adding 100 µl of 50 mM catechol in 100 mM potassium phosphate buffer (pH 7.0) to 50 µl enzyme extract. The absorbance was measured at 490 nm using a UV/Vis spectrophotometer before and after 90 min dark incubation at RT.

### 2.7 Phytohormones analysis

#### 2.7.1 Sample preparation

Ten milligrams of freeze-dried ground samples were used for metabolite extraction with 1 mL solvent mixture (methyl-*tert*-butyl ether:methanol: water, 6:3:1). The sample was vortexed 90 min at 4°C, then centrifuged for 15 min at 4°C at 3,500 × g. Four hundred µL of the supernatant was transferred to a new tube, dried down under a gentle stream of nitrogen, and resuspended in 200 µL of methanol.

#### 2.7.2 Standard curve

Phytohormones were quantified by establishing an external standard curve for each compound, A standard curve was generated by adding 40 mL of a phytohormone standards mix [Salicylic Acid (SA), Jasmonic Acid (JA), Abscisic Acid (ABA), Indole-3-acetic Acid (IAA), Jasmonoyl-isolucine (JA-Ile), Trans-Zeatin, 12-Oxo-phytodienoic Acid (OPDA), Indole-3-carboxylic Acid (ICA)] at a concentration of 10 g/mL to 760 mL of a 6:3:1 MTBE:methanol: water solution containing deuterated internal standards, and 2-fold serial dilutions were applied to the mixture. After being dried under nitrogen gas and reconstituted in 200 µL of methanol, 200 l aliquots were transferred to inserts for analysis.

#### 2.7.3 Liquid chromatography-mass spectrometry

Phytohormone quantitation was performed at the University of California, Riverside Metabolomics Core Facility as described previously (Sheflin et al., 2019). Briefly, analysis was conducted on a TQ-XS triple quadrupole mass spectrometer (Waters Corp., Milford, MA) coupled to an I-class UPLC system (Waters Corp., Milford, MA). Separations were carried out on a T3 C18 column (2.1 × 100 mm, 1.8 µM) (Waters Corp., Milford, MA). The mobile phases were (A) water and (B) acetonitrile, both with 0.1% formic acid. The flow rate was 300 µL/min, and the column was held at 45°C. The injection volume was 2 µL. The gradient was as follows: 0 min, 0.1% B; 1 min, 0.1% B; 6 min, 55% B; 7 min, 100% B; 8 min, 100% B; 8.5 min, 0.1% B; 13 min, 0.1% B. The MS was operated as described in the primary metabolite analysis section.

The MS was operated in an assigned reaction monitoring mode. Temperatures at the source and during desolvation were 150°C and 600°C, respectively. Desolvation gas flow was adjusted at 1100 L/hr, and cone gas to 150 L/hr. The collision gas flow rate was set at 0.15 mL/min. All gases were nitrogen except for the collision gas, which was argon. In the positive ion mode, the capillary voltage was 1 kV, while 2 kV in the negative ion mode. To check on the stability and effectiveness of the system, a quality control sample was created by pooling equal aliquots of each sample. Samples were examined in a random sequence.

### 2.8 Primary metabolites analysis

The frozen ground tissues conserved at -80 C were freeze-dried and ten milligrams of each sample were used for metabolite extraction with 1 mL solvent mixture (acetonitrile:methanol:isopropanol: water, 30:30:20:20). The sample was vortexed 60 min at 4°C, sonicated in an ice bath for 15 min, and then centrifuged for 15 min at 4°C at 16,000 × g.

Primary metabolite measurements and analyses were conducted at the University of California, Riverside Metabolomics Core Facility as described previously (Griffith et al., 2019). Metabolomic analysis was performed on a TQ-XS triple quadrupole mass spectrometer (Waters Corp., Milford, MA) coupled to an I-class UPLC system (Waters Corp., Milford, MA). Separations were carried out on a ZIC-pHILIC column (2.1 × 150 mm, 5 µM) (EMD Millipore, Burlington, MA). The mobile phases were (A) water with 15 mM ammonium bicarbonate adjusted to pH 9.6 with ammonium hydroxide and (B) acetonitrile. The flow rate was 200 µL/min, and the column was held at 50°C. The injection volume was 1 µL. The gradient was as follows: 0 min, 90% B; 1.5 min, 90% B; 16 min, 20% B; 18 min, 20% B; 20 min, 90% B; 28 min, 90% B.

The mass spectrometry was operated in a selected reaction monitoring mode. Source and desolvation temperatures were 150°C and 600°C, respectively. Desolvation gas was set to 1100 L/hr, and cone gas to 150 L/hr. All gases were nitrogen except the collision gas, which was argon. The capillary voltage was 1kV in positive ion mode and 2 kV in negative ion mode. The quality control sample, generated by pooling equal aliquots of each sample, was analyzed periodically to monitor system stability and performance.

## 3 Results

### 3.1 Changes in biochemical components in the cell wall

**Figure 2:**
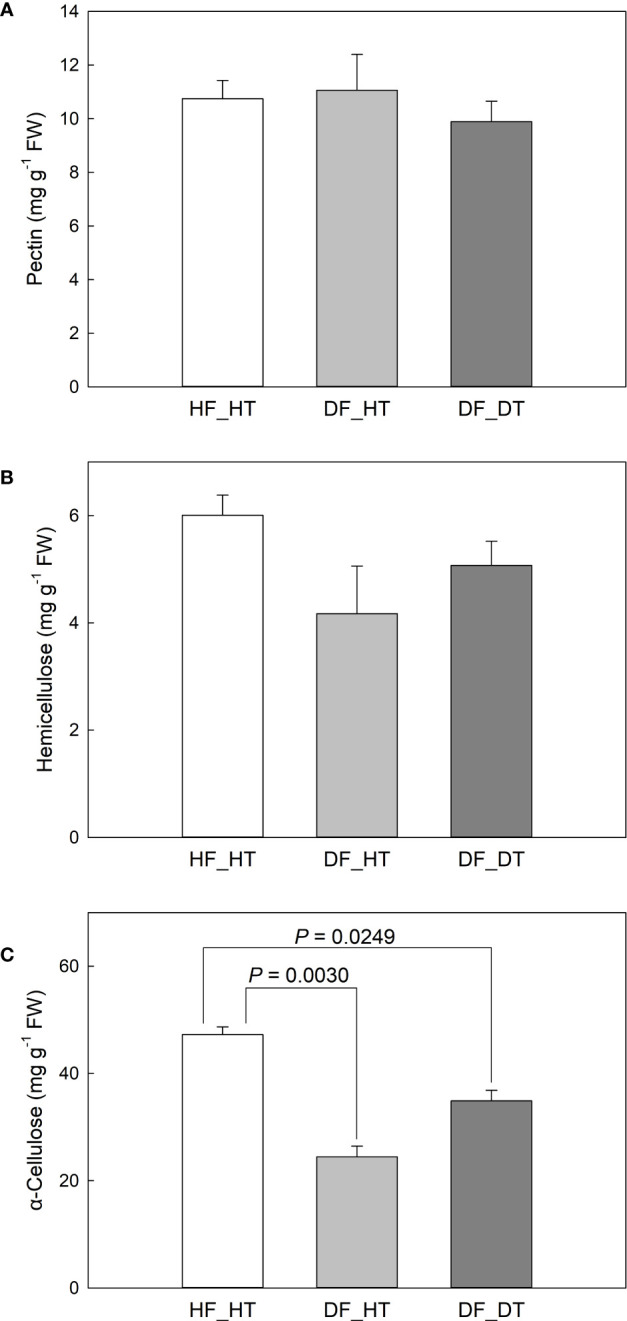
Satsuma mandarin (C. unshiu Marc. ‘Owari’) rind cell wall fractions: **(A)** pectin, **(B)** hemicellulose, and **(C)** α-cellulose. Vertical bars are the standard error of the means with four replications (n = 4). Significant differences between samples are indicated with the P-value.

Pre-harvest rind disorder in citrus fruits is a serious physiological disorder and influences fruit yield and storage quality. The study was carried out using ten-years-old Satsuma Oawri mandarin trees grafted on Carrizo rootstock and grown in a commercial orchard in San Joaquin Valley, California, USA. The rind tissues were collected from healthy tissue of healthy fruit (HF_HT), healthy tissue of damaged fruit (DF_HT), and damaged tissue of damaged fruit (DF_DT). Physiological and biochemical parameters were analyzed using pairwise comparisons between samples (HF_HT vs. DF_HT, HF_HT vs. DF_DT, and DF_HT vs. DF_DT) (**Supplementary Figure 1**). Plant cell walls mostly consist of pectin, which is a group of polysaccharides rich in galacturonic acid. In this study, we investigated pectin no significant difference was observed in healthy tissues compared to damaged tissues. (**Figure 2A**, **Supplementary Figure 1**). In order to ensure the specificity and adaptability of cell wall transformation in various cell types and conditions, pectins’ structural diversity and the diverse combinations of the enzymes responsible for pectin biosynthesis and modification play crucial roles in cell wall integrity (Shin et al., 2021). The results showed that hemicellulose accumulation was significantly higher in healthy tissues (HF_HT), followed by DF_DT and DF_HT (**Figure 2B**). The hemicellulose production was limited in damaged tissues, and it might lead to the degradation of cell wall components. Compared with healthy tissues (HF_HT), damaged fruit tissues (DF_HT and DF_DT) had lower α-cellulose content (*P* = 0.0030 and 0.0249, respectively), but had similar contents of pectin and hemicellulose (**Figure 2**). No significant difference was observed in the contents of cell wall components between DF_HT and DF_DT. Cellulase was involved in the breakdown of cellulose and hemicellulose, the development of the disorder, and the softening of fresh fruits (Lin et al., 2017; Chen et al., 2021). Therefore, all health tissues (HF_HT) showed higher cell wall integrity, and strength was decreased as the disorder appeared on the rind surface.

### 3.2 Cellular antioxidant activity

**Figure 3:**
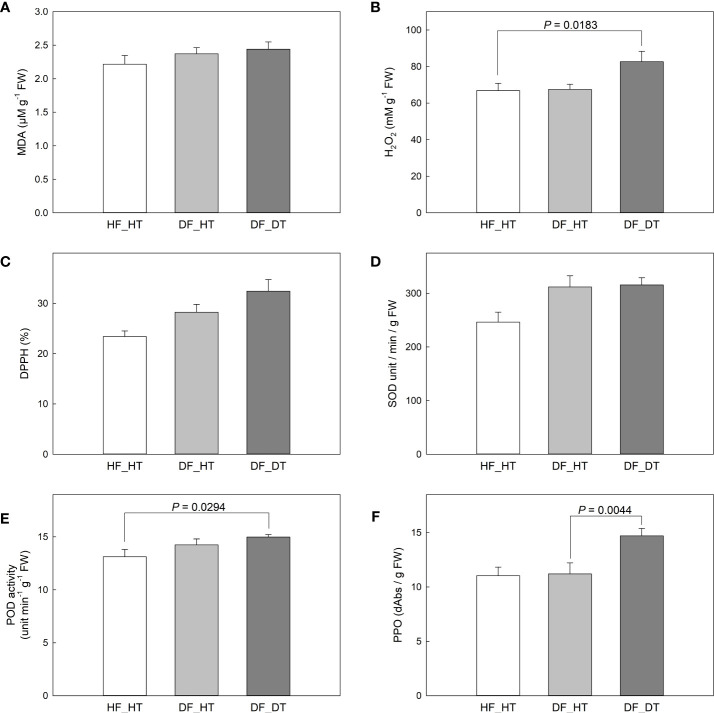
Changes of MDA content in Satsuma mandarin (C. unshiu Marc. ‘Owari’) rind: **(A)**, H2O2 content **(B)**, DPPH free radical scavenging activity **(C)**, SOD activity **(D)**, POD activity **(E)**, and PPO activity **(F)**. Vertical bars are the standard error of the means with four replications (n = 4). Significant differences between samples are indicated with the P-value.

The enzymes superoxide dismutase (SOD), catalase (CAT), peroxidase (POX), and ascorbate peroxidase (APX) have been associated with enzymatic antioxidant scavenging systems. Superoxide anion radicals are broken down into O_2_ and H_2_O_2_ by SOD enzymes, and H_2_O_2_ is subsequently detoxified by APX, POX, and CAT enzymes into either H_2_O or O_2_ (Mittler, 2002; Lee et al., 2012). In the present study, we investigated antioxidant activities to assess the oxidative stress associated with rind disorder. There was no significant difference in all paired comparisons between samples in the MDA content (**Figure 3A**). DF_DT had a higher H_2_O_2_ content compared to HF_HT (*P* = 0.0183), while DF_HT had similar content to that of HF_HT (**Figure 3B**). Total antioxidant activity measured by DPPH and SOD activity was not different between samples (**Figures 3C, D**), while POD and PPO activities were increased in DF_DT compared to HF_HT (*P* = 0.0294) and DF_HT (*P* = 0.0044), respectively (**Figures 3E, F**, **Supplementary Figure 1**).

### 3.3 ABA, JA, and OPDA content

**Figure 4:**
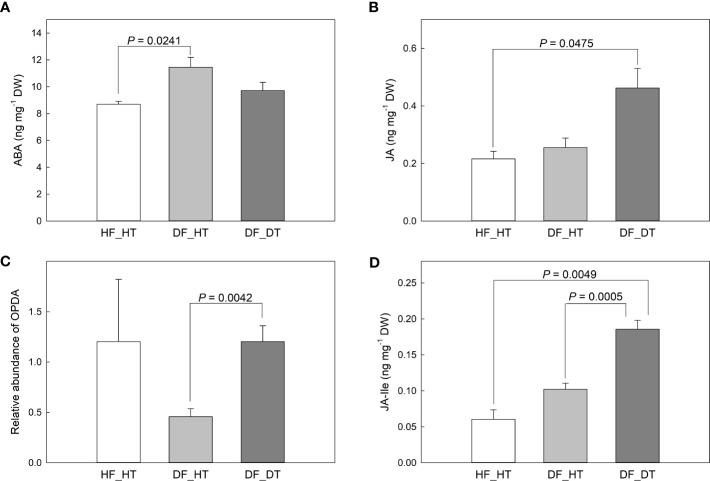
Contents of abscisic acid (ABA, **A**), jasmonic acid (JA, **B**), 12-oxo-phytodienoic acid (OPDA, **C**), and JA-isoleucine (JA-Ile, **D**) in Satsuma mandarin (C. unshiu Marc. ‘Owari’) rind. Vertical bars are the standard error of the means with four replications (n = 4). Significant differences between samples are indicated with the P-value.

**Figure 5:**
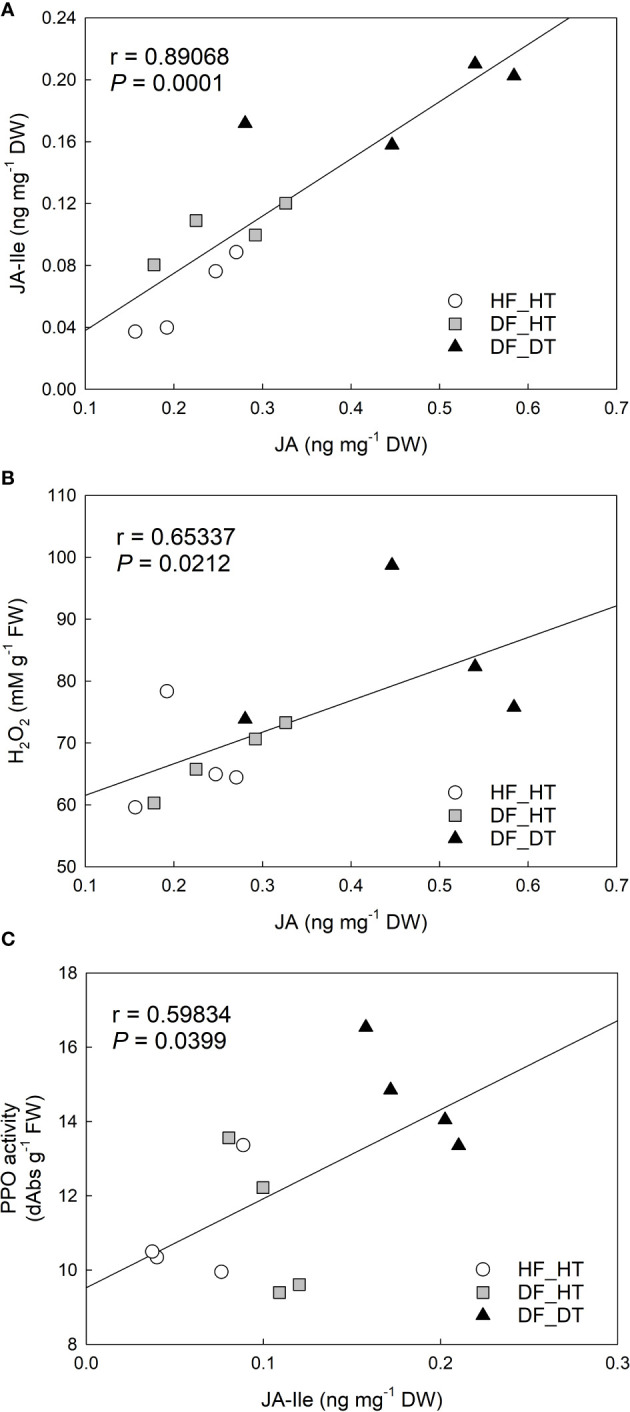
Correlation analysis between H2O2 content, PPO activity, and jasmonic acid (JA), JA-isoleucine (JA-Ile) in Satsuma mandarin (C. unshiu Marc. ‘Owari’) rind: **(A)** JA vs. JA-Ile; **(B)** JA vs. H2O2 content; **(C)** JA-Ile vs. PPO activity.

ABA and JA function in cell wall integrity and play a critical role in determining fruit ripening, metabolic activities, and cell wall degradation. We examined abscisic acid (ABA), jasmonic acid (JA), and 12-oxo-phytodienoic acid (OPDA) content in relation to rind disorder. The contents of ABA, JA, and its precursor (OPDA) and derivatives (JA-isoleucine, Ja-Ile) were shown in **Figure 4**. Compared to HF_HT, the ABA content was increased in DF_HT (*P* = 0.0241) but not in DF_DT (**Figure 4A**). DF_DT showed increased JA compared to HF_HT (*P* = 0.0475, **Figure 4B**) and increased OPDA abundance compared to DF_HT (*P* = 0.0042, **Figure 5C**). The highest content of JA-Ile was found in DF_DT (**Figure 4D**, **Supplementary Figure 1**). Overall, DF_DT had high contents of JA and its precursor and derivative.

### 3.4 Antioxidants correlation analysis

Correlation analysis was performed with parameters of cell damage, antioxidant enzymes, and hormones. There were significant relationships between H_2_O_2_ production, PPO activity, and JA and its derivative, JA-Ile. The content of JA was positively correlated to that of JA-Ile (r = 0.89068 and *P* = 0.0001, **Figure 5A**) and H2O2 production (r = 0.65337 and *P* = 0.0212, **Figure 5B**). JA-Ile displayed a significant positive relationship with PPO activity (r = 0.59834 and *P* = 0.0399, **Figure 5C**).

### 3.5 Metabolite profiling

**Figure 6:**
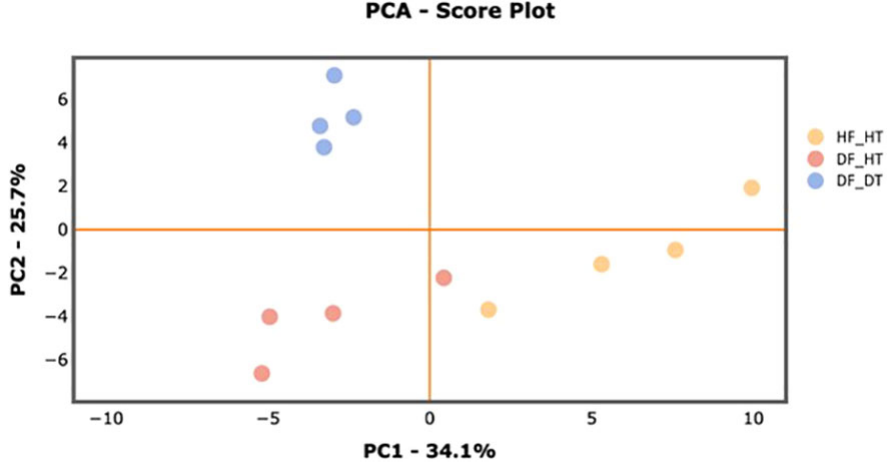
PCA score plot of primary metabolite profiles differentiating HF-HT (yellow), DF-HT (orange), and DF-DT (blue).

**Figure 7:**
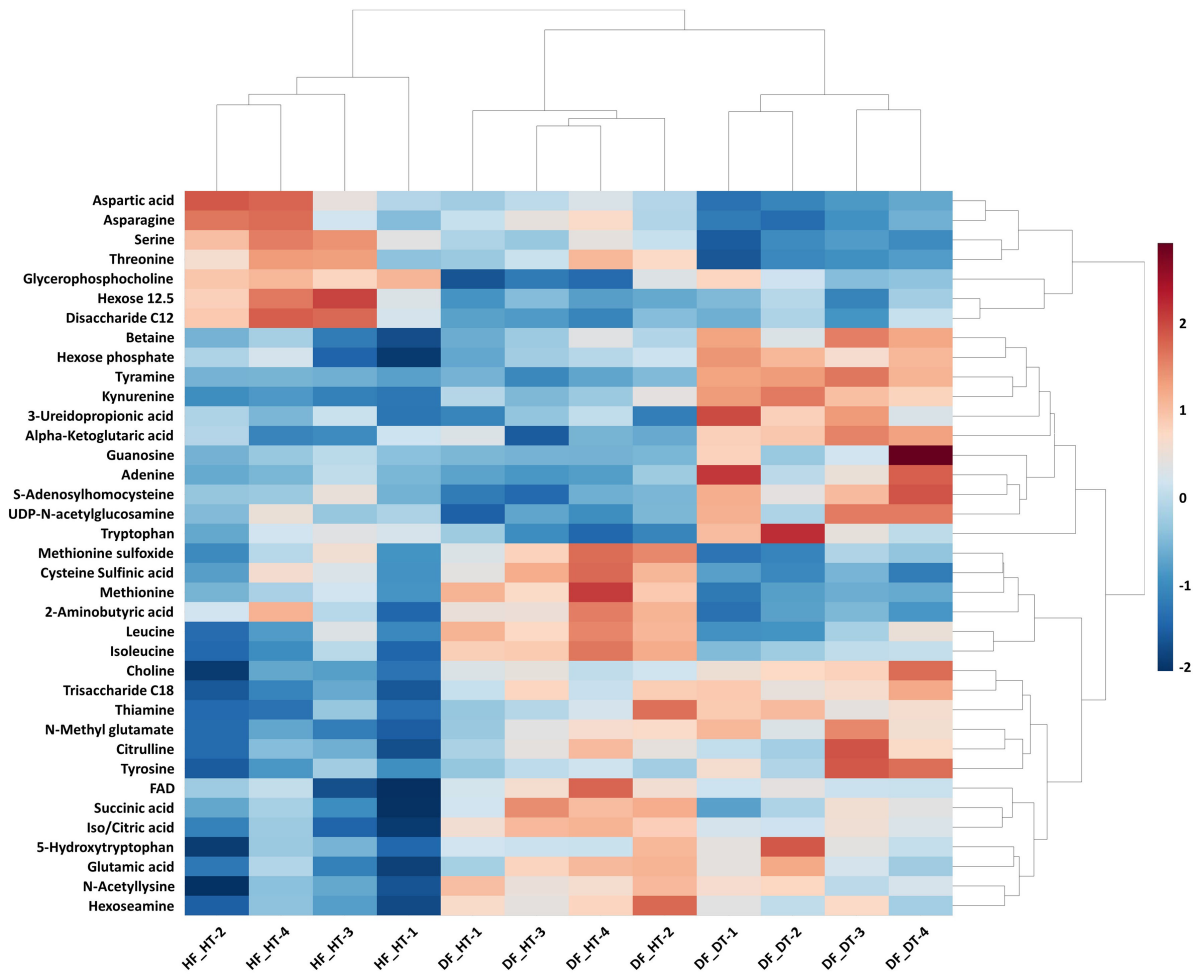
Hierarchical cluster heatmap of significant metabolites in Satsuma mandarin (C. unshiu Marc. ‘Owari’) rind. The row represents individual metabolites, and the column displays samples. Metabolites significantly decreased were displayed in blue, while metabolites significantly increased were displayed in red.

Many secondary metabolites, including flavonoids, alkaloids, coumarins, limonoids, carotenoids, phenol acids, and essential oils, are found in citrus fruits (Lv et al., 2015). Targeted metabolomics analysis was conducted to identify primary metabolites associated with rind disorder. In a principal component analysis (PCA) based on accumulated metabolites, the first two principal components separated all samples and explained around 60% of the total variation in the entire data set (**Figure 6**). The PC1 accounted for 34.1% of the variation distinguishing healthy and damaged fruit, whereas the PC2 accounted for 25.7% of the variation separating healthy and damaged tissues. The hierarchical cluster heatmap formed three distinguished clusters among samples, indicating rind disorder changed primary metabolite profiles not only between healthy and damaged fruit but also between healthy and damaged tissues in damaged fruit (**Figure 7**). Few significantly upregulated metabolites in DF_DT including betaine, hexose phosphate, tyramine, and kynurenine were recorded. A total of 76 metabolites were identified in Satsuma rind tissues, and the relative concentrations of 43 metabolites were significantly different across samples (**Table 1**). The main difference was found in amino acids, followed by compounds involved in the TCA cycle. The balance of endogenous hormones is essential for controlling source-sink communications between the TCA cycle, glycolysis, sucrose, and starch metabolism (Brenner, 1987; Ibáñezibá˜ibáñez et al., 2014). Most metabolites were significantly higher in DF_HT and/or DF_DT than in HF_HT. The relative concentrations of N-methyl glutamate, hexoseamine, amino acid, N-acetylcysteine, methionine, and tyramine were increased; while, flavin adenine dinucleotide, succinic acid, asparagine, threonine, and serine were decreased in DF_HT and DF_DT, respectively, compared to HF_HT. Twenty-four metabolites were significantly different between DF_HT and DF_DT. Among them, half were increased in DF_HT, and the other half were increased in DF_DT. Notably, Kynurenine and iso/citric acid were 1.32 and 1.56-fold higher in DF_HT and 2.19 and 1.26-fold higher in DF_DT than HF_HT, respectively. Tyramine was increased in DF_DT by 1.11 and 1.18-fold compared to HF_HT and DF_HT, respectively. In contrast, asparagine was 1.30 and 1.04-fold lower in DF_DT than HF_HT and DF_HT, respectively.

**Table 1:** Fold changes of differentially accumulated primary metabolites in Satsuma mandarin (C. unshiu Marc. ‘Owari’) rind.

Compound	Class	DF_HT/HF_HT	DF_DT/HF_HT	DF_DT/DF_HT
4-Guanidinobutyric_acid	Amino acid	0.24	0.30*	0.06
Tyramine	Amino acid	−0.07	1.11***	1.18***
Tyrosine	Amino acid	0.29	0.62**	0.33
Betaine	Methylation	0.28	0.64**	0.37*
Adenine	Purine	−0.11	0.56**	0.67**
Guanosine	Purine	−0.35	1.14*	1.49*
3-Ureidopropionic_acid	Pyrimidine	−0.06	0.40*	0.46*
myo-Inositol	Sugar alcohol	0.00	0.07*	0.07*
Hexose_phosphate	Sugar phosphate	0.20	0.55*	0.35
alpha-Ketoglutaric_acid	TCA cycle	−0.03	0.36*	0.38*
N-Acetyllysine	Acetyl amino acid	0.48**	0.40**	−0.08
5-Hydroxytryptophan	Amino acid	0.37*	0.44*	0.07
Glutamic_acid	Amino acid	0.18*	0.15*	−0.03
Isoleucine	Amino acid	0.38***	0.16*	−0.22**
Kynurenine	Amino acid	1.32***	2.19***	0.87**
Hexoseamine	Amino sugar	0.29**	0.20**	−0.09
Thiamine	B vitamin	0.43**	0.52**	0.09
Choline	Choline	0.39**	0.56***	0.16
N-Methylglutamate	Methyl amino acid	0.35***	0.45***	0.10
Glutathione	Oxidative stress	0.25*	0.27*	0.02
Trisaccharide_C18	Sugar	0.39***	0.46***	0.07
Isocitric_acid	TCA cycle	1.56**	1.26**	−0.30
Citrulline	Urea cycle	0.51*	0.56*	0.05
Cysteinesulfinic_acid	Amino acid	0.30*	−0.19	−0.48**
Leucine	Amino acid	0.41**	0.09	−0.32*
Methionine	Amino acid	0.40**	−0.14	−0.54***
Flavin adenine dinucleotide	Electron carrier	0.47*	0.33	−0.14
Methionine_sulfoxide	Oxidative stress	0.38*	−0.12	−0.50*
Succinic_acid	TCA cycle	0.38**	0.20	−0.17
S-Adenosylhomocysteine	Methylation	−0.23*	0.33*	0.56***
UDP-N-acetylglucosamine	Pyrimidine	−0.33*	0.37*	0.70**
Asparagine	Amino acid	−0.25	−1.30**	−1.04**
Threonine	Amino acid	−0.06	−0.37**	−0.31**
Aspartic_acid	Amino acid	−0.30*	−0.65**	−0.36*
Serine	Amino acid	−0.24**	−0.54***	−0.30**
Disaccharide_C12	Sugar	−0.19**	−0.15**	0.04
Hexose_at RT12.5	Sugar	−0.20**	−0.18**	0.03
Glycerophosphocholine	Choline	−0.79**	−0.33	0.46*
2-Aminobutyric_acid	Amino acid	0.20	−0.19	−0.39*
Lysine	Amino acid	0.31	−0.60	−0.91*
Tryptophan	Amino acid	−0.27	0.21	0.47**
Malic_acid	TCA cycle	0.23	−0.08	−0.31*

The values were calculated as log_2_ fold change. ^*,**,***^Significant at P< 0.05, 0.01, 0.001, respectively.

## 4 Discussion

Despite the fact that some physiological changes only affect the fruit’s rind and do not damage the edible core, they do reduce the market value of postharvest fruit because the citrus fruit’s exterior appearance is a key criterion for judging quality and a common consumer complaint (Magwaza et al., 2014). Therefore, in the present study, the rind tissues from healthy and damaged fruits were analyzed to investigate the causes of rind disorders and their impact on fruit quality. The MDA content showed that there was no significant variance in all paired comparisons between samples (**Figure 3A**). DF_DT had a higher H_2_O_2_ content compared to HF_HT (*P* = 0.0183), while DF_HT had similar content to that of HF_HT (**Figure 3B**). Ali et al. (2019) reported that excessive production of free radicals damages cell membranes and that the increased synthesis of H_2_O_2_ under low temperatures is considered harmful for fruits. Similarly, it is possible that the higher levels of H_2_O_2_ observed in the DF_DT areas of the rind in this study had a role in the development of rind damage. The lack of differences in MDA content, however, does not correspond with prior work that indicates that increased MDA level demonstrates more oxidative stress and is a crucial sign of cell membrane breakdown (Ali et al., 2019; Ali et al., 2020; Ali et al., 2021). Total antioxidant activity, measured by DPPH, and SOD activity, showed a non-significant difference between samples (**Figure 3**), while POD and PPO activities were increased in DF_DT compared to HF_HT (*P* = 0.0294) and DF_HT (*P* = 0.0044), respectively (**Figures 3E, F**). Furthermore, the membrane lipid ratio of fresh fruit cells changed during postharvest storage. PPO activity increased under stressful conditions, and this resulted in the formation of chemical barriers preventing further ROS from spreading. Polyphenol oxidases appear to have a role in plant immunity, and recent evidence suggests they may also be involved in other physiological processes (Taranto et al., 2017) PPO is responsible for the oxidation of antioxidants, subsequently decreasing the antioxidant activity of cold-stored fruit and leading to the induction of chilling injury and loss of membrane integrity during cold storage (Lee et al., 2012; Khefifi et al., 2020; Xu et al., 2021). The polyphenol oxidase enzyme found in fruits causes the enzymatic browning reaction. Enzymatic browning is mostly observed in fruits during harvest, shipping, storage, and processing, it affects the nutritional and sensory qualities of fruits (Queiroz et al., 2008; Moon et al., 2020).

In the present study ABA, JA, and its precursor (OPDA) and derivative (Ja-Ile) were evaluated in their relationship to rind damage (**Figure 4**). Compared to HF_HT, the ABA content was increased in DF_HT (*P* = 0.0241) but not in DF_DT. Alférez et al. (2005) reported that the ABA level in the Navel orange fruit rind influences the frequency of certain physiological rind disorders such as post-harvest pitting and chilling injury, but there was no clear role of ABA with rind damage in this study. Among phytohormones, JA, as well as its precursors and derivatives, are crucial in mediating plant responses and defenses to biotic and abiotic challenges (Wang et al., 2021). (Magwaza et al., 2019) reported that the highest concentration of ABA catabolites, including 7-OH-ABA and DPA, were found in fruit that was present inside the tree canopy and shaded, and these fruits also responded more strongly to rind disorder. The highest content of JA-Ile was found in DF_DT. Overall, DF_DT had high contents of JA and its precursor and derivative. The balance between plant development and defense is controlled by the interaction of JA with several other plant hormones (Wang et al., 2021). The results depicted a significant relationship between H_2_O_2_ production, PPO activity, and JA and its derivative, JA-Ile (**Figure 5**). Reactive oxygen species and subsequent defense mechanisms are induced by exogenous JA and methyl jasmonate (MJ) in plant organs. Additionally, it triggers signal transmission, activates several defense genes, and causes the buildup of secondary metabolites (Ho et al., 2020). Several oxylipin jasmonic acid (JA) derivatives constitute the jasmonate hormones, which are crucial regulators of plant defense. Among these, it has been demonstrated that the conjugate jasmonoyl-isoleucine (JA-Ile) interacts with the jasmonate co-receptor complex directly to control reactions to jasmonate signaling (Schuman et al., 2018). The content of JA was positively correlated to that of JA-Ile (r = 0.89068 and *P* = 0.0001), and H_2_O_2_ production (r = 0.65337 and *P* = 0.0212). Many physiological processes in plant growth and development, including the modulation of plant responses to biotic and abiotic stressors, are known to be possessed by jasmonic acid (JA) and its precursors and derivatives (Bertini et al., 2018; Bertini et al., 2019). MeJA triggers the accumulation of secondary metabolites and induces oxidative stress in plant cells (Ho et al., 2020).

In the present study, several metabolites were significantly upregulated in DF_HT and/or DF_DT than in HF_HT. The relative concentrations of N-methyl glutamate, hexosamine, amino acid, N-acetylcysteine, methionine, and tyramine were increased; while flavin adenine dinucleotide, succinic acid, asparagine, threonine, and serine were decreased in DF_HT and DF_DT, respectively, compared to HF_HT. Lu et al. (2017) reported more than 850 metabolites in CK and RD peels at the fruit development stage. The intermediates of primary metabolism are involved in sugar, organic acid, and amino acid metabolism intermediates. Only plants and microorganisms can produce L-tyrosine (Tyr), an aromatic amino acid (AAA) needed for protein synthesis in all living things. Tyr also acts as a precursor in plants for a variety of specialized metabolites with physiological functions like antioxidants, electron carriers, attractants, and defense molecules (Schenck and Maeda, 2018). Additionally, many amino acids serve as precursors in the manufacture of other nitrogenous substances including nucleotides, phytohormones, or secondary metabolites. Furthermore, under conditions of elevated photorespiration, the serine concentration may be significant. Lastly, stress greatly increases the pools of all amino acids (Jacoby et al., 2011; Schenck and Maeda, 2018). The significant changes in the main metabolites of amino acids, organic acids, and sugars in fruit rind. Twenty-four metabolites were significantly different between DF_HT and DF_DT. Among them, half were secondary metabolites that differ either quantitatively or qualitatively across species, populations, and tissues and organs of the same species, as well as between individuals within a population (del Valle et al., 2015; Rao et al., 2021). The primary and secondary metabolites of *C. unshiu* have been identified as flavonoids and terpenoids, which included limonoids, carotenoids, monoterpenes, and sesquiterpenes. Chalcone synthase and isomerase convert p-coumaroyl-CoA into naringenin to produce flavonoids involved in the plant defense response (Ledesma-Escobar et al., 2019; Kim et al., 2022). The most significant metabolites in DF_DT including betaine, hexose phosphate, tyramine, and kynurenine were up-regulated (**Figure 7**). Tyramine-derived hydroxycinnamic acid amines (HCAAT) are naturally occurring secondary metabolites produced in a wide range of plant species. It belongs to the neutral, water-insoluble compounds and plays an important role in plant defense mechanisms, growth, and development (Leonard et al., 2022). Tyramine release is decreased by long-term environmental stresses like heat or oxidative stress, which promotes the activation of cytoprotective genes (Mittler, 2002; De Rosa et al., 2019; Pervaiz et al., 2022). Glycine betaine helps plants develop and survive by reversing metabolic disorders caused by severe stress (Annunziata et al., 2019). Furthermore, Melatonin (MT) is a novel, highly effective plant growth regulator that is tested on a variety of crops to withstand abiotic stress. Melatonin and its precursor tryptophan (Try) considerably boost the ability of plants to survive under stress (Sadak and Ramadan, 2021). However, the physiological and ecological importance of secondary metabolites established from tryptamine is often still unknown. Tryptophan (Trp) is a crucial precursor of secondary metabolites in plants. The relationship between the production of secondary metabolites generated from tryptamine and the activity of the enzymes suggests that TDC is a regulatory component in the biosynthesis of secondary metabolites in plants (Hildebrandt et al., 2015; Sadak and Ramadan, 2021).

## 5 Conclusion

In the present work, we investigated the most emerging rind disorder in Satsuma ‘Owari’ Mandarin. The main difference was found in amino acids, followed by compounds involved in the TCA cycle. The hormone results depicted that the damaged fruit tissues have a higher level of jasmonic acid, OPDA, and JA-isoleucine than undamaged tissue. In addition, damaged fruits (DF_HT and DF_DT) showed lower cellulose concentrations than healthy fruit tissues (HF_HT), however, had similar contents of pectin and hemicellulose. Therefore, the cell wall may be involved in various cellular processes and cell wall integrity was influenced, and fruit damage was started. Fruit that was more prone to rind disorder had a distinct rind biochemical profile than healthy tissues. Overall, DF_DT had high contents of JA and its precursor and derivative, it is known that MeJA triggers the accumulation of secondary metabolites and induces oxidative stress in plant cells. JA may be one of the key hormones that play significant roles in the initiation of pre-harvest citrus rind disorder. Management of the JA and its derivatives level in the fruits might be the key to controlling the citrus pre-harvest rind disorder. However, more studies are needed to test this hypothesis.

## Data availability statement

The original contributions presented in the study are included in the article/**Supplementary Material**. Further inquiries can be directed to the corresponding author.

## Author contributions

SP and AR conducted experiments and collecting data, MH metabolic analysis, TP and SP drafted the manuscript; DO, ML, and AE conceived the ideas, review the manuscript, and provided project funding support.
