# Supplementary material for: Metabolomic analyses provide insights into the preharvest rind disorder in Satsuma Owari Mandarin
Source: Front Plant Sci. 2023 Sep 26;14:1263354. doi: 10.3389/fpls.2023.1263354 (PMC10562707; doi:10.3389/fpls.2023.1263354)
Supplement: Supplementary file 1 [file DataSheet_1.pdf]

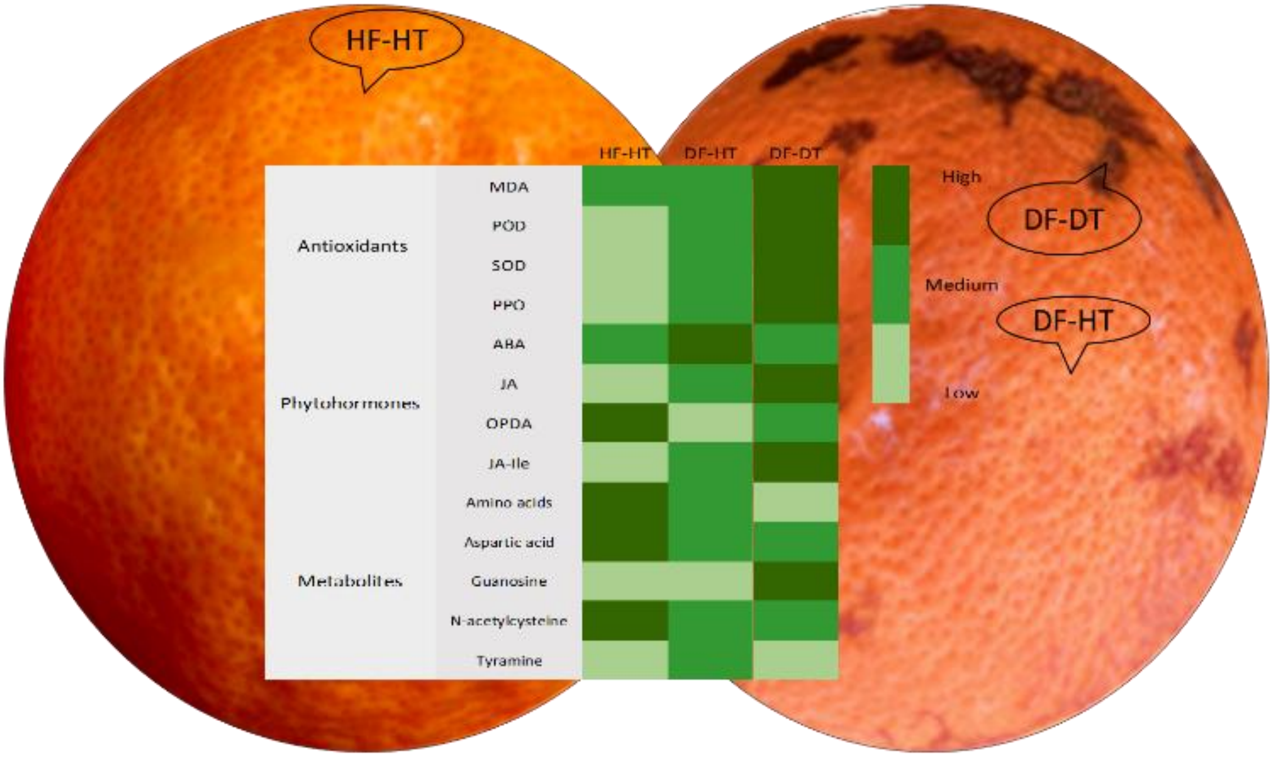


**Supplementary Figure. 1** The schematic diagram shows the sampling tissues from HF-HT, DF-DT, and DF-DT. Heatmap presents, the antioxidant content, Phytohormones concentration, and significant metabolites in studies samples (light to dark green color transition shows the lower to higher values in each component)
